# Two novel mutations in *PRPF3* causing autosomal dominant retinitis pigmentosa

**DOI:** 10.1038/srep37840

**Published:** 2016-11-25

**Authors:** Zilin Zhong, Min Yan, Wan Sun, Zehua Wu, Liyun Han, Zheng Zhou, Fang Zheng, Jianjun Chen

**Affiliations:** 1 Department of Ophthalmology of Shanghai Tenth People’s Hospital, and Tongji Eye Institute, Tongji University School of Medicine, Shanghai, China; 2 Department of Medical Genetics, Tongji University School of Medicine, Shanghai, China; 3 Center for Gene Diagnosis, Zhongnan Hospital of Wuhan University, Wuhan, China; 4 National Laboratory of Biomacromolecules, Institute of Biophysics, Chinese Academy of Sciences, Beijing, China

## Abstract

Retinitis pigmentosa (RP) is a heterogeneous set of hereditary eye diseases, characterized by selective death of photoreceptor cells in the retina, resulting in progressive visual impairment. Approximately 20–40% of RP cases are autosomal dominant RP (ADRP). In this study, a Chinese ADRP family previously localized to the region between D1S2819 and D1S2635 was sequenced via whole-exome sequencing and a variant c.1345C > G (p.R449G) was identified in *PRPF3*. The Sanger sequencing was performed in probands of additional 95 Chinese ADRP families to investigate the contribution of *PRPF3* to ADRP in Chinese population and another variant c.1532A > C (p.H511P) was detected in one family. These two variants, co-segregate with RP in two families respectively and both variants are predicted to be pathological. This is the first report about the spectrum of *PRPF3* mutations in Chinese population, leading to the identification of two novel *PRPF3* mutations. Only three clustered mutations in *PRPF3* have been identified so far in several populations and all are in exon 11. Our study expands the spectrum of *PRPF3* mutations in RP. We also demonstrate that *PRPF3* mutations are responsible for 2.08% of ADRP families in this cohort indicating that *PRPF3* mutations might be relatively rare in Chinese ADRP patients.

Retinitis pigmentosa (RP) refers to a heterogeneous set of hereditary retinal degenerative disorders which are responsible for the blindness of more than 1.5 million people worldwide and affect about 1 in 1000 people in China. RP is characterized by a selective death of photoreceptors that are light-sensing cells in the retina, resulting in progressive visual impairment^1^,^2^,^3^.There are three modes of Mendelian inheritance in RP—autosomal-dominant RP (ADRP), autosomal-recessive RP (ARRP), and X-linked RP (XLRP)^1^. Approximately 20–40% of RP cases are ADRP and mutations in over 20 genes are known to cause ADRP. Amongst ADRP causative genes are an unusual class— pre-mRNA splicing genes^4^— eight of which are ubiquitous core snRNP proteins (*PRPF3*, PRPF8, *PRPF3*1, PRPF4, SNRNP200, and PRPF6) and splicing factors (RP9 and DHX38). Mutations in those genes ubiquitously expressed and essential for splicing, have so far been reported to cause a disease that displays retina-specific phenotype^5^.

*PRPF3* (RP18, OMIM 601414) gene spans approximately 32 kb at chromosome 1q21^6^, contains 16 exons and encodes a protein of 683 amino acids in length with a calculated molecular weight of 77 kDa^7^, which is a human homologue of the yeast U4/U6-associated splicing factor Prp3. Only three clustered *PRPF3* mutations, c.1482C > T (p.T494M), c.1478C > T (p.P493S) and c.1466C > A (p.A489D), have been identified thus far in RP in several populations. The mutation T494M is the most frequently detected substitution in *PRPF3* while P493S occurs rather sporadically^5^. All the mutations are in the exon11 (c.1427–1526) of *PRPF3* and previous surveys failed to identify mutations outside of this exon. Therefore, only the region c.1427–1526 was screened for testing *PRPF3* mutations in some reported studies^8^,^9^. In this work, we identified two novel *PRPF3* mutations, c.1345C > G (p.R449G) and c.1532A > C (p.H511P), in two Chinese families with ADRP. Furthermore, our study demonstrates that *PRPF3* mutations are responsible for 2.08% of ADRP families in our cohort indicating that *PRPF3* mutation might be relatively rare in Chinese patients with ADRP.

## Results

### Clinical evaluations of ADRP families

**Figure 1:**
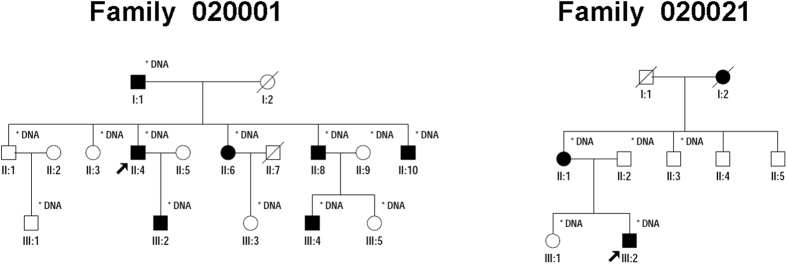
Pedigrees of Family 020001 and Family 020021. Probands are pointed out by arrow. Circles indicate females, and squares, males. Filled symbols indicate affected patients, and empty symbols, healthy controls. Individuals who were genotyped are marked with an asterisk.

The pedigree of Family 020001 indicates a dominant inheritance pattern of three generations (Fig. 1). Medical history of all the affected individuals shows that early onset of night blindness is at age 4 to 10 years old, with subsequent loss of far peripheral vision after 20 years. Clinical details of that family were previously described^10^. Additional 95 Chinese families had a primary diagnosis of RP based on clinical descriptions provided by the referring clinicians at the time of enrollment. The inheritance pattern of those RP families is AD (Fig. 1).

### Mutation analysis

**Figure 2:**
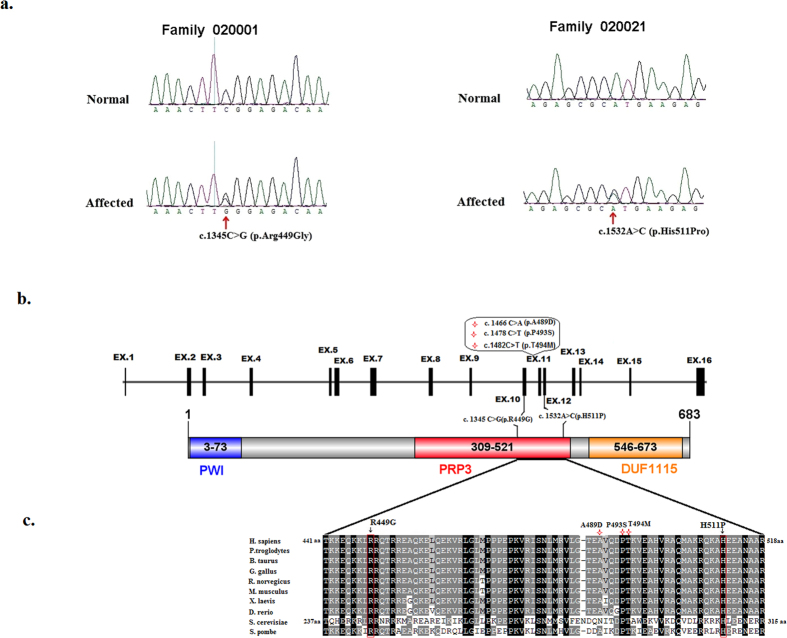
Mutations identified in Family 020001 and Family 020021. (**a**) Sequence chromatograms showing the identified mutations and their wild type form. (**b**) Schematic representation of the linear location of our identified *PRPF3* mutations and previously reported mutations (red star) in context of genome (upper) and protein (below). (**c**) Orthologous protein sequence alignment of *PRPF3* from human (H. sapiens), chimpanzees (P. troglodytes), cattle (B. taurus), Chicken (G. gallus), Norway rat (R. norvegicus), house mouse (M. musculus), African clawed frog (X. laevis), zebra fish (D. rerio), yeast (S. cerevisiae), and Pombe yeast (S. pombe). Completely conserved residues across all species aligned are shaded with black. Residues boxed in red show the conservation of residues identified mutations in this study.

We selectively performed whole-exome sequencing (WES) on the proband (II: 4) of Family 020001 previously mapped to the region between D1S2819 and D1S2635^10^ (95.53Mb-159.17 Mb). An average of 9.71 Gb of sequence data were obtained, and 99.94% of bases originated from the targeted exome, resulting in a mean coverage of 115-fold. More than 98.94% of the targeted exons were covered more than 10-fold. We employed the following filters to filter the exome results: total variants → heterozygous variants → variants that are absent in the 1000 Genomes Project, 1000G_ASN, esp6500si_all, and dbSNP137 → variants that are coding or splicing → variants that are predicted to be pathogenic. Following the filteration procedures described above, only one heterozygous missense variant in *PRPF3* (NM_004698: exon10: c.1345C > G: p.R449G) was detected to be located within the linkage region between D1S2819 and D1S2635^10^, which would lead to the change of amino acid from Arginine to Glycine at residue 449 (p.R449G). Sanger sequencing confirmed this variant (Fig. 2a). The variant was predicted to be probably damaging with a score of 0.980 by PolyPhen-2, a score of 0.05 by SIFT, and a score of −6.698 by PROVEAN. Intra-familial co-segregation analysis of Family 020001 further revealed that this heterozygous missense variant was present in all the affected and absent in normal family members, which further confirmed that the variant was a potential ADRP causing mutation for Family 020001.

To investigate the contribution of *PRPF3* to ADRP in Chinese population, Sanger sequencing of *PRPF3* was performed in the probands of additional 95 Chinese ADRP families. We identified another novel variant c.1532A > C in exon12 of *PRPF3* gene in Family 020021 (Fig. 2a), which would lead to the change of amino acid from Histidine to Proline at residue 511 (p.H511P). This variant co-segregated in the family with RP and was predicted to be probably damaging with a score of 1.000 by PolyPhen-2, a score of 0.00 by SIFT, and a score of −9.785 by PROVEAN.

These two mutations, c.1345C > G (p.R449G) and c.1532A > C (p.H511P), were further confirmed to be absent in 200 unrelated ethnically matched healthy controls as well as in database of probably benign variation. Both mutations are absent either in existing database of disease-causing mutations or in the reported literatures and thus considered to be novel.

**Figure 3:**
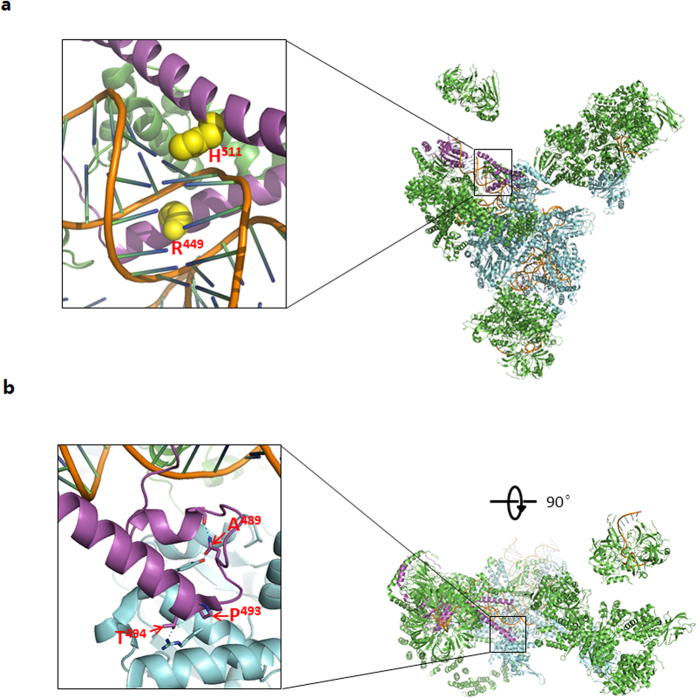
Structural model of the wild type Prp3 in the spliceosomal U4/U6.U5 tri-snRNP from S. cerevisiae. The residues (words in red) shown in the figures are completely conserved with the mutated residues in human *PRPF3* by MSA. The positions of those residues in human *PRPF3* indicated in the figures, which correspond to the same residues in Prp3 of Saccharomyces cerevisiae S288c (NP_010761.3) respectively by MSA of *PRPF3* from Homo sapiens to yeast. (**a**) Both amino acid H (yellow) and R (yellow) play a key role in Prp3 (purple)-U4/U6 snRNA duplex (brown) interaction.(**b**) The residues (pointed out by red open arrows)of Prp3 (purple) are important in protein-protein interaction.

Multiple orthologous sequence alignment (MSA) revealed that p.R449G and p.H511P were highly conserved from human to yeast (Fig. 2c). The residues R^449^ and H^511^ in human *PRPF3* correspond to the residues R^245^ and H^308^ in Prp3 of Saccharomyces cerevisiae S288c (NP_010761.3), respectively, by MSA of *PRPF3* from Homo sapiens to yeast. Prp3 structure from PDB (Protein Data Bank): 3JCM (Cryo-em Structure of the yeast spliceosomal U4/U6.U5 Tri-snrnp) shows that amino acids R^245^ and H^308^ of Prp3 play a key role in Prp3 binding to RNA duplex^11^ suggesting that changes in those two residues may impair the structure and function of spliceosomal U4/U6.U5 Tri-snrnp (Fig. 3a).

## Discussion

*PRPF3* gene is one of pre-mRNA splicing genes and located in the region 150.29–150.33 Mb on chromosome 1q21^6^.Though *PRPF3* is essential for splicing and ubiquitously expressed in almost every cell of the body, mutations in the gene have so far been reported to cause a disease that displays retina-specific phenotype RP. So far, only three clustered *PRPF3* mutations, c.1482C > T (p.T494M)^7^,^9^,^12^,^13^,^14^,^15^,^16^, c.1478C > T (p.P493S)^7^,^9^ and c.1466C > A (p.A489D)^17^, have been reported in RP in several populations.

T494M is a common mutation of *PRPF3* in ADRP as it has also been found in English, Danish, Spanish, American, Japanese, Korean, and Swiss ADRP families. P493S occurs in a sporadic RP case from UK or Germany and in a Caucasian ADRP family from American. A489D was reported in a Spanish ADRP family (Table 1). All these three mutations are in the exon 11 (c.1427–1526) of *PRPF3*, for which only the region c.1427–1526 was screened for testing *PRPF3* mutations in reported studies including the original linkage paper about Family 020001 of this study, because previous surveys failed to find mutations outside of this exon^8^,^9^. The mutation c.1345C > G (p.R449G) is in the exon 10 and c.1532A > C (p.H511P) is in the exon 12, outside of the region frequently analyzed for detecting *PRPF3* mutation. In our study, the identification of two novel *PRPF3* mutations expands the spectrum of *PRPF3* mutations in RP and suggests that more *PRPF3* mutations might be found if the region outside of the exon 11 is screened. *PRPF3* mutations associated with RP are reported in different populations but there is no report about *PRPF3* mutation in Chinese population. This is the first report of the identification of *PRPF3* mutation in the Chinese population. Total of 96 Chinese ADRP families were recruited in our study and two *PRPF3* mutations were identified in two of the families. *PRPF3* mutations are responsible for 2.08% of ADRP families in this cohort indicating that *PRPF3* mutations might be relatively rare in Chinese patients with ADRP.

*PRPF3* gene encodes a 683aa protein^7^, which is a human homologue of the yeast U4/U6-associated splicing factor Prp3^18^. *PRPF3* protein contains one PWI motif^19^, one PRP3 domain and a DUF1115 domain (Fig. 2b)^20^. Three previously reported mutations and two novel mutations in this study all locate in PRP3 domain (Fig. 2b). MSA revealed that those five residues mutated in RP patients were highly conserved across all the species (Fig. 2c). Structural analysis of the wild type Prp3 in the yeast spliceosomal U4/U6.U5 Tri-snrnp (PDB: 3JCM) showed that the yeast counterparts of human Prp3 residues R^449^ and H^511^ buried into two successive grooves of RNA duplex, suggesting their necessity in U4/U6 snRNA duplex interaction^11^ (Fig. 3a), which suggest that two novel mutations R449G and H511P probably impair binding of *PRPF3*- RNA duplex because both glycine and proline have propensity to break α-helical structure of *PRPF3*. In contrast, yeast counterparts of human *PRPF3* residuesT^494^, P^493^ and A^489^ are clustered in the C-terminal conserved region, which play an important role in protein-protein interaction^5^ (Fig. 3b), which is mentioned the previous report. Our study may provide alternative insights into the relevant pathogenesis for RP.

In summary, we, for the first time, reported *PRPF3* mutation spectrum in the Chinese population and demonstrated that *PRPF3* mutations are responsible for 2.08% of ADRP families in this cohort indicating that *PRPF3* mutation might be relatively rare in Chinese patients with ADRP. Two novel mutations, c.1345C > G (p.R449G) and c.1532A > C (p.H511P), were identified in our study, which expands the spectrum of *PRPF3* mutations in RP. Structure analysis provides alternative insights into the etiology of RP. However, future research is still warranted to establish the pathogenic mechanism underlying how novel *PRPF3* mutations identified in this study would cause RP.

## Methods

### Study Subjects and Clinical Examinations

The Institutional Review Board (IRB) of Tongji Eye Institute of Tongji University School of Medicine (Shanghai, China) and the Center for Gene Diagnosis, Zhongnan Hospital of Wuhan University (Wuhan, China) approved this study and the whole procedure of this study adhered to the tenets of the Declaration of Helsinki. All participating family members provided informed written consent that has been endorsed by the respective IRBs and is consistent with the tenets of the Declaration of Helsinki.

A three-generation Chinese Family 020001 with ADRP was recruited for this study. The routine and ophthalmologic examination were performed on all patients including visual acuity tests, the condition of the fundus, the extent of the visual field, and flash electroretinogram after informed consent form was obtained. Genotyping and linkage analysis were completed by Yuan *et al*.^10^. 7 affected and 6 unaffected individuals from this family were collected 5 ml blood samples. We also recruited another cohort of 200 unrelated and ethnically matched individuals, and an additional 95 ADRP families. Total genomic DNA was isolated with DNA extraction kits (TianGen, Beijing, China) according to the manufacturer’s protocol.

### Whole-exome sequencing and data analysis

Genomic DNA (5 μg) from the proband (individual II: 4) of Family 020001 was sent to Biotechnology Corporation (Shanghai, China) for whole-exome capture followed by sequencing. The whole exome was captured using Agilent SureSelect Human All Exon Kit according to the manufacturer’s instructions. Briefly, samples were first prepared as Illumina sequencing libraries, which were then enriched for the desired target according to the Illumina Exome Enrichment protocol. Then, each captured library was loaded on a HiSeq 2500 platform, where paired-end sequencing was conducted with read lengths of 100 bp, providing an average coverage depth of at least 100× for each sample. Data were aligned to UCSC Genome Browser build hg19 by the Burroughs Wheeler Aligner. Local realignment was performed with the Genome Analysis Toolkit IndelRealigner and variants were called with SAM tools. Variants were filtered against 1000 Genomes Project, 1000G_ASN, esp6500si_all, and dbSNP137. Copy-number variations (CNVs) and structural variants from WES data were also assessed and bioinformatic prediction was based on BAM files analyzed by the Integrative Genomics Viewer (IGV) and CoNIFER.

### Sanger sequencing and *in silico* analysis

In all members of Family 020001 with available DNA samples, the mutation was validated by Sanger sequencing as previously described^15^. The primers of all coding exons including flanking sequence of RPRF3 were designed by the web-based version of the Primer3 program (Table S1). Samples from another cohort of 200 unrelated ethnically matched controls and the probands of additional 95 ADRP families were also sequenced to detect variations in *PRPF3*.

Both variants were analyzed to determine the likelihood of pathogenicity with the following procedure. First, existing databases of disease-causing mutations (e.g. the Human Gene Mutation Database at http://www.hgmd.org) were searched for previous reports of the variant. Secondly, databases of probably benign variation such as dbSNP (http://www.ncbi.nlm.nih.gov/snp/), the 1000 Genomes database 9 (http://browser.1000genomes.org/index.html), and the NHLBI Exome Sequencing Project database (http://evs.gs.washington.edu/EVS) were also examined to determine if the variant was found in healthy controls. Third, evolutionary conservation of the amino acids region including mutation was assessed via the GeneDoc program (www.cris.com/~ketchup/genedoc.shtml) through alignment of the *PRPF3* orthologous protein sequences of the following species: Homo sapiens (NP_004689.1), Pan troglodytes (NP_001233355.1), Bos taurus (NP_001039516.1), Gallus gallus (NP_001026561.1), Rattus norvegicus (NP_001102029.1), Mus musculus (NP_001303680.1), Xenopus laevis(NP_001085273.1). Danio rerio (NP_991311.2), Saccharomyces cerevisiae S288c (NP_010761.3) and Schizosaccharomyces pombe 972h- (NP_594560.1). Fourth, SIFT (Sorting Intolerant from Tolerant, http://www.sift.jcvi.org/), PolyPhen-2 (Polymorphism Phenotyping v2, http://www.genetics.bwh.harvard.edu/pph2/) and PROVEAN (Protein Variation Effect Analyzer, http://provean.jcvi.org/) online servers were used to detect the potential pathogenic impacts of the mutation. Fifth, the positions of the amino acids found mutated in RP, corresponds to their positions in yeast Prp3 (NP_010761.3) respectively by sequence alignment of *PRPF3* from Homo sapiens and S. cerevisiae. The high resolution Cryo-em structure of the yeast spliceosomal U4/u6.u5 Tri-snrnp (PDB 3JCM) was used for analysis the potential effect of those mutations.

## Additional Information

**How to cite this article**: Zhong, Z. *et al*. Two novel mutations in *PRPF3* causing autosomal dominant retinitis pigmentosa. *Sci. Rep.* **6**, 37840; doi: 10.1038/srep37840 (2016).

**Publisher's note:** Springer Nature remains neutral with regard to jurisdictional claims in published maps and institutional affiliations.

## Supplementary Material

Supplementary Table S1

## Figures and Tables

**Table 1:** List of mutations of *PRPF3* linked to ADRP.

Exon	Nucleotide change	amino acid change	Inheritance	Phenotype	Samples with *PRPF3* mutation	Reference
Exon 10	c. 1345C > G	p. Arg449Gly	AD	RP	a Chinese family	This study
Exon 11	c. 1466A > C	p.Ala489Asp	AD	RP	a Spanish family	Gamundi *et al*.^17^
Exon 11	c. 1478C > T	p. Pro493Ser	AD	RP	a sporadic case from UK or Germany	Chakarova *et al*.^7^
Exon 11	c. 1478C > T	p. Pro493Ser	AD	RP	a Caucasian family from U.S.A.	Sullivan *et al*.^9^
Exon 11	c. 1482C > T	p. Thr494Met	AD	RP	two English families, one Danish family and two sporadic cases from UK or Germany	Chakarova *et al*.^7^
Exon 11	c. 1482C > T	p. Thr494Met	AD	RP	a Spanish family	Marti’nez-Gimeno *et al*.^13^
Exon 11	c. 1482C > T	p. Thr494Met	AD	RP	a Japanese family	Wada *et al*.^16^
Exon 11	c. 1482C > T	p. Thr494Met	AD	RP	a Caucasian family from U.S.A.	Sullivan *et al*.^9^
Exon 11	c. 1482C > T	p. Thr494Met	AD	RP	a Swiss family	Vaclavik *et al*.^12^
Exon 11	c. 1482C > T	p. Thr494Met	AD	RP	a France family	Audo *et al*.^15^
Exon 11	c. 1482C > T	p. Thr494Met	AD	RP	a Korean family	Kim *et al*.^14^
Exon 12	c. 1532A > C	p. His511Pro	AD	RP	a Chinese family	This study
