# Corrigendum: Two novel mutations in *PRPF3* causing autosomal dominant retinitis pigmentosa

**DOI:** 10.1038/srep45248

**Published:** 2017-03-28

**Authors:** Zilin Zhong, Ming Yan, Wan Sun, Zehua Wu, Liyun Han, Zheng Zhou, Fang Zheng, Jianjun Chen

Scientific Reports 6: Article number: 3784010.1038/srep37840; published online: 11 25 2016; updated: 03 28 2017

The original version of this Article contained a typographical error in the spelling of the author Ming Yan, which was incorrectly given as Min Yan.

Additionally, in the original version of this Article, Ming Yan was incorrectly affiliated with “Center for Gene Diagnosis, Zhongnan Hospital of Wuhan University, Wuhan, China”

The correct affiliation is listed below:

Department of Ophthalmology, Zhongnan Hospital of Wuhan University, Wuhan, China

These errors have now been corrected in the PDF and HTML versions of this Article.

