# Supplementary material for: Two novel mutations in PRPF3 causing autosomal dominant retinitis pigmentosa
Source: Sci Rep. 2016 Nov 25;6:37840. doi: 10.1038/srep37840 (PMC5122955; doi:10.1038/srep37840)
Supplement: Supplementary Table S1 [file srep37840-s1.pdf]

Two novel mutations in *PRPF3* causing autosomal dominant retinitis pigmentosa

Zilin Zhong[1, 2, +], Min Yan[3, +], Wan Sun[1, 2], Zehua Wu[1, 2], Liyun Han[1, 2], Zheng Zhou[4], Fang Zheng[3, *], and Jianjun Chen[1, 2, *]

Table S1. Primers used for amplification and sequence analysis of human PRPF3.

Primers	Forward (5’>3)	Reverse (5’>3’)	Amplicon Length (bp)
PRPF3_EX02	AGTATTGAGTCCTGTTTGAGCT	GGTGACTCAGTATGGTAGCC	428
PRPF3_EX03	TCAAGGTAAACAGGTTGCTCT	AGACAAAGCTGCCAATTATTTCT	587
PRPF3_EX04	GGAGGTGAGTTTTAGGCAGGA	TACAGACCCATGCACCTTTTG	545
PRPF3_EX05-06	TCCAGAGTCTCAATCATCACACT	GCCAGATCAGTAACATTCAAAGG	847
PRPF3_EX07	CTCAAGTGATCCCTCTGCCTT	TCACGAAACCATCCTTACTCCA	632
PRPF3_EX08	TGGAAGGTAGAGGTTGCTGTT	CAGGACCTTGTGTACAGTTACT	499
PRPF3_EX09	GAACATAGCGTCTGTCAGCTG	TGGGTAATGCTGCTCTAGAGA	398
PRPF3_EX10	TTTTATTGCTGTCCGGGCC	ACCAGAGTGTTGTATGATCAGT	668
PRPF3_EX11-12	GCTCTTTCACTAGATGCTGTGA	AGCTCTTGATCCACACTAGGG	825
PRPF3_EX13-14	AGTAAATACCTCTCCCTGACTCT	GGCTCAAAGTCTAAACGGGTC	892
PRPF3_EX15	TCCCAAAGTGCCTAGGACAAG	CATTCCCGTTTCCAAGTCTCA	680
PRPF3_EX16	CTGGGCACATGTCTCACAAAT	TCACCTGTAGACCCAAACTCT	521

Note: All primers were amplified using a touchdown protocol beginning at 64℃, decreasing by 0.5℃ each cycle, until finishing at a final annealing temperature of 57°℃.
